# Congenital adrenal hyperplasia due to 11-beta-hydroxylase deficiency: description of a new mutation, R384X

**Published:** 2016-09-30

**Authors:** Audrey Mary Matallana-Rhoades, Juan David Corredor-Castro, Francisco Javier Bonilla-Escobar, Bony Valentina Mecias-Cruz, Liliana Mejia de Beldjena

**Affiliations:** 1 Universidad del Valle, Cali, Colombia; 2 Hospital Universitario del Valle, Cali, Colombia; 3 Instituto Cisalva, Universidad del Valle, Cali, Colombia; 4 Fundación SCISCO, Cali, Colombia; 5 Fundación Clínica Valle del Lili, Cali, Colombia; 6 Fundación Clínica Infantil Club Noel, Cali, Colombia

**Keywords:** Adrenal hyperplasia congenital, hyperplasia adrenal glands, mutation, adrenocorticotropic hormone, Virilism, 11-beta-hydroxylase deficiency

## Abstract

**Case Description:**

It is presented the phenotype of a new compound heterozygous mutation of the genes R384X and Q356X encoding the enzyme of 11-beta-hydroxylase

**Clinical Findings:**

Severe virilization, peripheral hypertension, and early puberty.

**Treatment and Outcome:**

Managed with hormone replacement therapy (corticosteroid) and antihypertensive therapy (beta-blocker), resulting in the control of physical changes and levels of arterial tension.

**Clinical Relevance:**

According to the phenotypic characteristics of the patient, it is inferred that the *R384X* mutation carries an additional burden on the *Q356X* mutation, with the latter previously described as a cause of 11-beta-hydroxylase deficiency. The description of a new genotype, as in this case, expands the understanding of the hereditary burden and deciphers the various factors that lead to this pathology as well as the other forms of congenital adrenal hyperplasia (CAH), presenting with a broad spectrum of clinical presentations. This study highlights the importance of a complete description of the patient's CAH genetic profile as well as their parents' genetic profile.

## Abstract (es)

**Descripción del Caso:**

Se presenta el fenotipo de una nueva mutación heterocigota compuesta en los genes Q356X y R384X que codifican la enzima 11-beta-hidroxilada

**Hallazgos Clínicos:**

Virilización severa, pubertad precoz periférica e hipertensión.

**Tratamiento y Resultados:**

Manejo con terapia de reemplazo hormonal con corticoide y antihipertensivo con beta-bloqueador con lo que se logró controlar los cambios físicos y los niveles de tensión arterial.

**Relevancia Clínica:**

Según las características fenotípicas del paciente se infiere que la mutación R384X acarrea una carga adicional a la mutación Q356X, esta última descrita como causa de deficiencia de 11-beta-hidroxilasa. La descripción de nuevos genotipos, como en este caso, permite ampliar la comprensión de la carga hereditaria y descifrar los diversos factores que llevan a que esta patología, así como las demás formas de hiperplasia suprarrenal congénita (HSC), se presenten con un amplio espectro de cuadros clínicos. Esto permite resaltar la importancia de una descripción completa del perfil genético del paciente con HSC y de sus padres.

## Introduction 

Congenital adrenal hyperplasia (CAH) is a group of enzymatic disorders characterized by defects in one of the steps in cortisol production 1; the second most common cause of CAH is 11-beta-hydroxylase deficiency (6-8% of cases of CAH) 2, with a prevalence of 1 per 100,000 births 3, and is found mainly among the Moroccan Jewish population. CAH is clinically characterized by virilization accompanied by hypertension secondary to sodium retention and increased volume 1.

After the isolation of DNA encoding 11-beta-hydroxylase located on chromosome 8q21, a pair of genes was identified: *CYP11B1*, associated with the expression of 11-beta-hydroxylase, and *CYP11B2*, associated with the expression of aldosterone synthase 4. The *CYP11B1* and *CYP11B2* genes are closely related due to their relevance in the synthesis of corticosterone and aldosterone, respectively. Since their discovery, several mutations in the *CYP11B1* gene that are associated with 11-beta-hydroxylase deficiency have been described, and the vast majority of these mutations have a noticeable demographic or geographical distribution: the mutation *p*.*R448H* described in Jewish immigrants from Morocco 5; the mutations *g.4671_4672insC* and *g.2791G>A* described in Brazilians 6; the mutations *p.Q356X* (*c.1066C>T, rs146124466*) and *p.G379V (c.1136G>T)* described in Tunisians and persons of African origin 3,7; and more recently, the mutations *p.E67fs* and *p.R448H* described in Croatian families 8.

The following describes a clinical case of congenital adrenal hyperplasia due to 11-beta-hydroxylase deficiency in which a genetic mutation not previously described in the literature was identified.

## Clinical case

The patient was 2 years and 9 months of age, African descendant, with an assigned male sex, and he and his family were originated from Buga, Valle del Cauca, Colombia. The patient first attended the pediatric endocrinology service in May 2012 because of the appearance of pubic hair, acne, axillary odor, increased penis size, and high stature. Within the patient's background, it was found that he was born full term, had an adequate birth weight, and was the second pregnancy of a mother who had 2 deliveries. The patient's mother underwent prenatal care and did not show any complications during the pregnancy; her only relevant background was the presence of an unoperated umbilical hernia, a history of respiratory illness, and exposure to cigarette smoke. There was no reported exposure to toxic substances or relevant family history.

### Clinical findings 

The patient appeared to have a higher chronological age, presenting with blood pressure (BP) levels greater than the 99^th^ percentile, weight and height greater than 2 standard deviations (SD) for his age, the presence of macro- and microcomedones in his forehead and cheeks, growth of his penis, nonpalpable testes in the scrotum or the inguinal canal, glandular hypospadias, and hyperpigmentation of his genitals.

### Diagnostic evaluation 

Given the clinical findings, the suspected diagnosis was CAH, and thus, the laboratory tests described in Table 1 were requested. Imaging and complementary studies showed normal adrenal glands, the presence of an uterus, the absence of female genitals, a bone age of 10 years, and a 46XX karyotype. The adrenocorticotropic hormone (ACTH) levels were not tested due to administrative problems; however, the reported findings were considered sufficient to corroborate the diagnosis of CAH due to 11-beta-hydroxylase deficiency with a compromise to sexual differentiation by virilization (Prader stage V).

**Table 1.** Laboratory tests of the patient with congenital adrenal hyperplasia due to 11-beta-hydroxylase deficiency

Analysis	Result
17-OH-progesterone (ng/dL)	5,200
Dehydroepiandrosterone sulfate (mcg/dL)	61.5
Aldosterone (100 pg/dL)	
Renin (pg/dL)	<1.6
Cortisol (nmol/L)	21.5
Testosterone (mg/dL)	1.66
Sodium	141
Potassium	3.7
Chlorine	101
Desoxycorticosterone	High
Androstenedione	High

**Figure 1.**
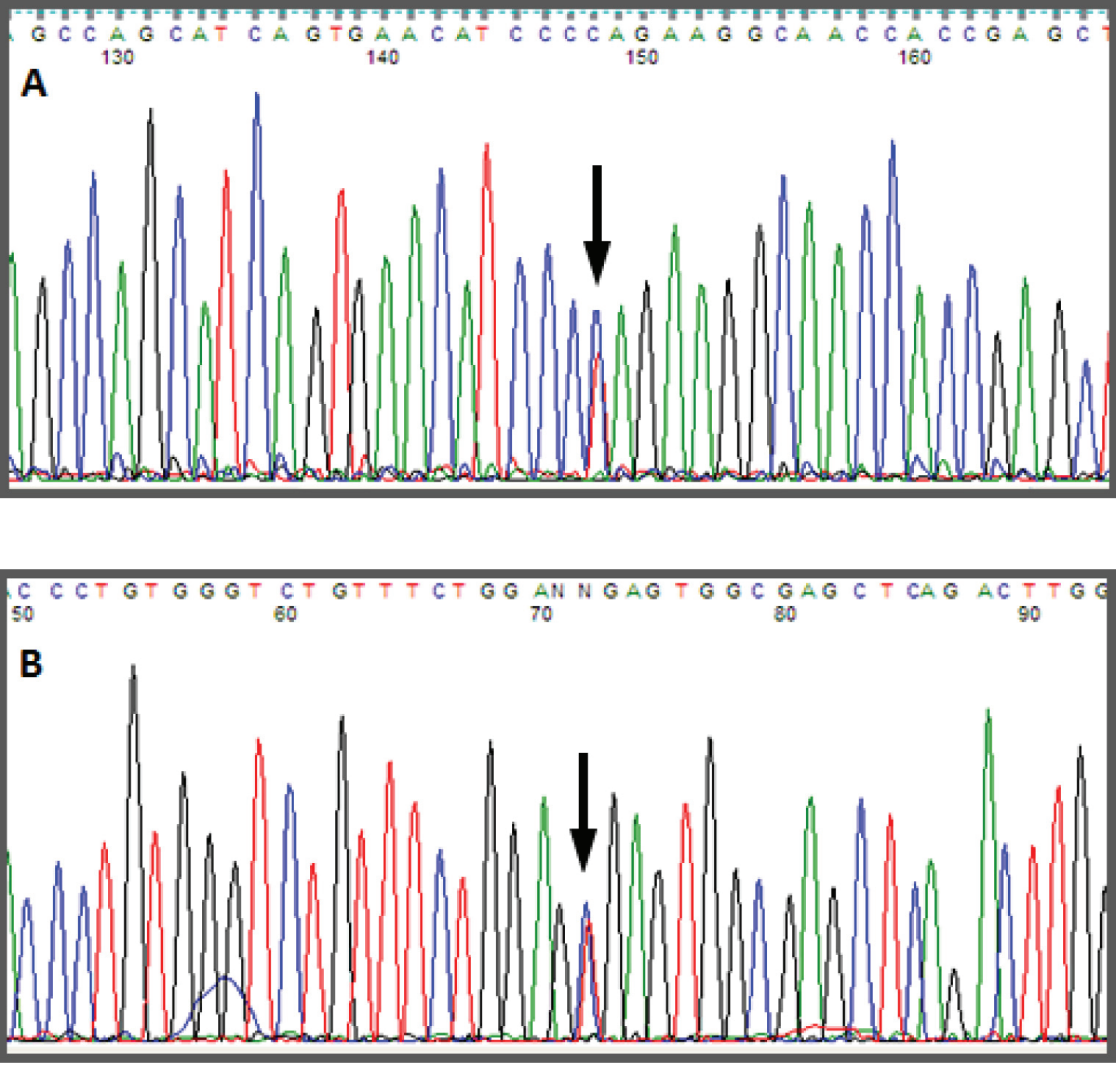
Mutations in the *CYP11B1* gene in codons (A) *Q356X* and (B) *R384X*.

**Figure 2.**
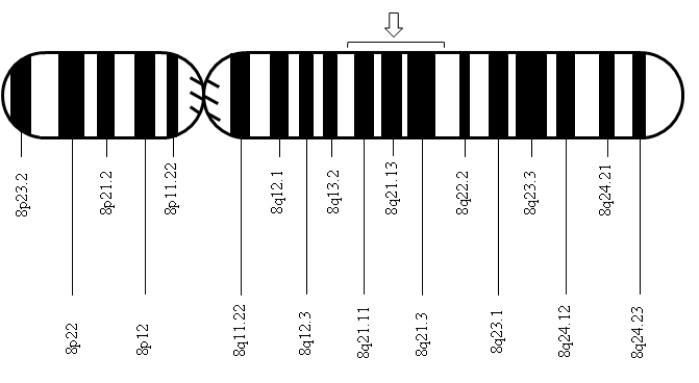
Localization of the *CYP11B1* gene mutation on chromosome 8q21.

**Figure 3.**
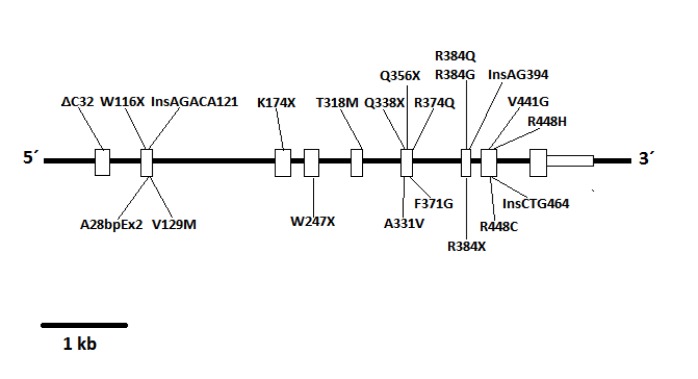
Localization of the newly described mutation, *R384X*, of the *CYP11B1* gene on chromosome 8q21.

Automatic sequencing was used to analyze the exon and intron regions neighboring the *CYP11B1* gene from genomic DNA of the patient and the mother after informed consent was obtained. A *Q356X* heterozygous mutation was found in exon 6 of the *CYP11B1* gene, which has previously been described in patients with an 11-beta-hydroxylase deficiency, and an *R384X* heterozygous alteration was found in exon 7 that results in a premature stop codon, causing the formation of a truncated protein without biological activity (Figs. 1-3). The *Q356X* heterozygous mutation was inherited from the patient's mother.

### Therapeutic intervention 

Once CAH was diagnosed, the complexity and consequences that the pathology could have on the patient's development were explained to the parents; they decided to keep their son's sex allocation as male. Management with prednisone 14 mg/m^2^/day was started to decrease the stimulus of ACTH and curb the production of 11-desoxycorticosterone acetate (DOCA), seeking to control the patient's BP. Propranolol was also prescribed to control BP, initially at a dose of 4 mg/Kg/day and finally at a dose of 40 mg/day due to lack of treatment adherence (TA) and not achieving BP goals. After the reduction of acne and pubic hair as well as a decline in the levels of 17-OH progesterone, a management scheme of prednisone 5 mg/day was decided upon.

### Follow-up and results 

The pharmacological management of the patient was limited by low TA, causing the patient to need hospitalization a year after his first consultation, as he presented with hypertensive urgency associated with a headache. After this hospitalization, TA was improved, and pharmacological management was optimized (propranolol was withdrawn and the patient continued with prednisone). Follow-up examination did not reveal an electrolyte disorder; the Tanner stage and bone age remained stable, and the levels of 17-OH progesterone dropped from 52.00 to 28.00 ng/dL after 1 year and 3 months of treatment. The patient was last assessed 2 years after his first visit, and a decrease in acne was observed; however, the adult penis and Tanner stage 3 persisted. 

## Discussion

Congenital adrenal hyperplasia is a group of diseases characterized by the absence of different enzymes involved in the synthesis of adrenal steroids due to mutation of the different genes that encode them, which leads to a hypersecretion of corticotropin-releasing hormone (CRH) and ACTH 9. Its prevalence worldwide is 1 per 100,000 people 3, without a sex predilection 1.

Congenital adrenal hyperplasia encompasses a diverse group of pathologies, each determined by a different enzymatic disorder and by measuring different degrees of penetrance. One of the enzymes affected is 11-beta-hydroxylase, which is responsible for the conversion of 11-desoxycorticosterone to corticosterone (both hormones possessing mineralocorticoid activity and being precursors to aldosterone) and 11-deoxycortisol to cortisol 10.

The clinical picture of CAH is defined by the absence of cortisol, leading to excessive secretion of ACTH and an accumulation of androgens and DOCA, and thus, patients present with alterations in sexual development, such as ambiguous genitalia in women, a large penis for their age in men, early puberty, or high stature; additionally, the accumulation of mineralocorticoids can lead to the development of hypertensive disorders and severe types of hydroelectrolytic imbalances of unclear pathophysiology 1,2,11,12. In the case presented, almost complete virilization, a high stature, and hypertensive disorder were found, without an alteration of the hydroelectrolytic state. 

The physiopathology of CAH is characterized by high levels of DOCA and 11-deoxycortisol in the blood biochemistry as well as a corresponding decrease in the levels of circulating aldosterone and cortisol. Additionally, a high level of ACTH is also found, resulting from the low cortisol level. Moderately high levels of adrenal androgens and testosterone as well as sometimes a moderate elevation of 17-OH progesterone can also be associated with CAH. The decreased secretion of renin caused by the negative feedback due to increased mineralocorticoid levels is due to the increase in DOCA ^1,2^. Interestingly, the patient presented with normal levels of cortisol and aldosterone, high levels of 17-OH progesterone, suppression of renin secretion, and increased levels of androstenedione and DOCA, which guided the patient's diagnosis. The levels of 11-deoxycortisol and ACTH were not measured due to availability problems in the region. 

The genetic diagnosis was performed based on the description of a mutation in the gene *CYP11B1* 10, which has been previously described in a variety of studies 3,5,6-8. Once a case is identified, the genetic characterization of both of the parents is important. In this case study, the *Q356X* mutation, which has been previously described in a homozygous state in patients of African origin (particularly from Tunisia) 3, 7 was identified in a heterozygous state in both the mother and child; additionally, an *R384X* mutation, which has not been previously described in the literature, was identified in exon 7 in a heterozygous state in only the child. The *R384X* mutation creates a premature stop codon, resulting in the presence of a truncated protein without biological activity, which would have a significant burden and would be responsible for allowing the development of the pathology despite the *Q356X* mutation being in a heterozygous state.

The treatment of CAH consists of hormone replacement therapy, which supplements glucocorticoids to reduce the synthesis of ACTH and its stimulus at the adrenal level 12. Sometimes, specific hypertension management is required, many times with potassium-sparing diuretics 1. In the case under discussion, management began with prednisone, and an adequate response in the levels of 17-OH progesterone was observed. However, the poor TA forced additional management with propranolol, which required multiple dose adjustments and finally required a dose of 40 mg/day. Many of the limitations in the control of symptoms were the result of poor adherence by the patient's mother to the disease management, which is why the absence of TA to monotherapy with glucocorticoids could be attributed. Management with the antihypertensive was discontinued once adequate compliance with the steroid scheme by the mother was assured.

## Conclusion

We describe the case of a patient who clinically presented with accumulation of DOCA and had a late diagnosis of CAH, which was not associated with a hydroelectrolytic imbalance. The patient had an assigned male sex from the appearance of his external genitalia but was karyotyped as 46XX at 2 years of age. Additionally, a mutation, *R384X,* not previously described in the literature was found, highlighting the importance of this case. The absence of testicles in the apparent scrotum and inguinal canal after physical examination is worth noting; had it been identified from the time of birth, it could have led to an early diagnosis and avoided the dilemma of dealing with a mismatch between chromosomal and assigned sex at a later age. Thus, the importance of physical examination and relevant study in patients with apparent anorchia must be emphasized. 

Finally, the various mutations associated with the 11-beta-hydroxylase deficiency have been associated with ethnic groups with similar demographic characteristics, resulting in data characterizing these alterations. The description of a new mutation in Colombia opens up the possibility of descriptions of the genetic pattern of other patients with 11-beta-hydroxylase deficiency to determine whether it is a mutation with a founder effect that is unique in the Colombian population. 
